# Can Haptic Stimulation Enhance Music Perception in Hearing-Impaired Listeners?

**DOI:** 10.3389/fnins.2021.723877

**Published:** 2021-08-31

**Authors:** Mark D. Fletcher

**Affiliations:** ^1^University of Southampton Auditory Implant Service, Faculty of Engineering and Physical Sciences, University of Southampton, Southampton, United Kingdom; ^2^Institute of Sound and Vibration Research, Faculty of Engineering and Physical Sciences, University of Southampton, Southampton, United Kingdom

**Keywords:** neuroprosthetic, cochlear implant, hearing aid, tactile aid, electro-haptic stimulation, pitch, multi-sensory, sensory substitution

## Abstract

Cochlear implants (CIs) have been remarkably successful at restoring hearing in severely-to-profoundly hearing-impaired individuals. However, users often struggle to deconstruct complex auditory scenes with multiple simultaneous sounds, which can result in reduced music enjoyment and impaired speech understanding in background noise. Hearing aid users often have similar issues, though these are typically less acute. Several recent studies have shown that haptic stimulation can enhance CI listening by giving access to sound features that are poorly transmitted through the electrical CI signal. This “electro-haptic stimulation” improves melody recognition and pitch discrimination, as well as speech-in-noise performance and sound localization. The success of this approach suggests it could also enhance auditory perception in hearing-aid users and other hearing-impaired listeners. This review focuses on the use of haptic stimulation to enhance music perception in hearing-impaired listeners. Music is prevalent throughout everyday life, being critical to media such as film and video games, and often being central to events such as weddings and funerals. It represents the biggest challenge for signal processing, as it is typically an extremely complex acoustic signal, containing multiple simultaneous harmonic and inharmonic sounds. Signal-processing approaches developed for enhancing music perception could therefore have significant utility for other key issues faced by hearing-impaired listeners, such as understanding speech in noisy environments. This review first discusses the limits of music perception in hearing-impaired listeners and the limits of the tactile system. It then discusses the evidence around integration of audio and haptic stimulation in the brain. Next, the features, suitability, and success of current haptic devices for enhancing music perception are reviewed, as well as the signal-processing approaches that could be deployed in future haptic devices. Finally, the cutting-edge technologies that could be exploited for enhancing music perception with haptics are discussed. These include the latest micro motor and driver technology, low-power wireless technology, machine learning, big data, and cloud computing. New approaches for enhancing music perception in hearing-impaired listeners could substantially improve quality of life. Furthermore, effective haptic techniques for providing complex sound information could offer a non-invasive, affordable means for enhancing listening more broadly in hearing-impaired individuals.

## Introduction

Cochlear implants (CIs) recover hearing for severely-to-profoundly hearing-impaired individuals by electrically stimulating the cochlea. They deploy an array of up to 22 microelectrodes, replacing the approximately 3,500 hair cells that transfer sound to the brain in normal-hearing listeners. Despite the fact that only limited sound information can be provided through this small number electrodes, CIs have been remarkably successful at recovering access to speech in quiet listening conditions (Zeng et al., 2008). However, CI users typically have impaired speech recognition in background noise (Fletcher et al., 2019, 2020b), as well as substantially reduced sound-localization accuracy (Dorman et al., 2016; Fletcher et al., 2020a) and music enjoyment (McDermott, 2004; Drennan et al., 2015). Hearing-aid (HA) users and other hearing-impaired listeners have similar performance limitations, though typically to a lesser extent (Looi et al., 2008; Dorman et al., 2016; Miller et al., 2016).

Several studies have recently shown that haptic stimulation can enhance CI listening by allowing access to sound features that are poorly transferred through electrical CI stimulation (see Fletcher, 2020; Fletcher and Verschuur, 2021). This “electro-haptic stimulation” can substantially improve speech-in-noise performance (Huang et al., 2017; Fletcher et al., 2018, 2019, 2020b), sound localization (Fletcher and Zgheib, 2020; Fletcher et al., 2020a), and melody recognition (Huang et al., 2019; Luo and Hayes, 2019), as well as discrimination of basic sound features such as pitch (Fletcher et al., 2020c). The impressive performance found in studies of haptic sound-localization and haptic enhancement of pitch discrimination suggests that it could also assist HA users (Fletcher and Zgheib, 2020; Fletcher et al., 2020a, c). There is also evidence that haptic stimulation can improve timbre discrimination (Russo et al., 2012) and music appreciation (Nanayakkara et al., 2009) in HA users. Music represents the biggest challenge for signal processing as it is often an extremely complex acoustic signal that contains several simultaneous harmonic and inharmonic sounds. Progress in enhancing music perception could therefore have strong implications for enhancing listening in the complex auditory environments in which hearing-impaired listeners often struggle to understand speech, such as busy offices, classrooms, or restaurants.

This review will focus on the use of haptic stimulation to enhance music perception in hearing-impaired listeners. Most people in the deaf community report being involved in music activities (Darrow, 1993) and music is central to many significant events, such as weddings and funerals, as well as to media, such as film. It is an important part of interactions with children (Hallam, 2010), can strongly influence the mood of films and the audience’s connection to the characters (Hoeckner et al., 2011), and can even bias shopping habits (North et al., 1999). As will be discussed, music perception is highly limited in many hearing-impaired listeners. This review first assesses the limits of music perception in hearing-impaired listeners, the suitability of the tactile system for transferring musical signals, and the evidence that audio and haptic inputs are integrated in the brain. It then discusses the existing haptic systems for enhancing music perception, the evidence of their utility, and the signal-processing approaches that could be deployed on future devices. Finally, it reviews the cutting-edge technologies that could be utilized for haptic enhancement of music perception.

## Is Haptic Stimulation Suitable for Enhancing Music Perception?

### Music Perception in Hearing-Impaired Listeners

When considering whether a haptic system might enhance music perception in hearing-impaired listeners, it is important to first establish the limits of music listening when hearing is impaired. It has been reported that, after a CI is implanted, only around 15% of adults enjoy listening to music (Philips et al., 2012) and around 70% are disappointed by how music sounds (Mirza et al., 2003). On a 10-point visual analog scale, CI users rated their musical enjoyment at 8.7 on average prior to hearing loss and at just 2.6 after implantation (Mirza et al., 2003). Low music appreciation has also been found for HA users, with those that have the most severe hearing loss reporting the lowest music appreciation (Looi et al., 2019). Some hearing-impaired listeners describe music as sounding “dissonant,” “out-of-tune,” “fuzzy,” and “tinny” (Uys et al., 2012; Jiam et al., 2017).

Numerous studies have explored which of the auditory features within musical pieces can be effectively extracted by hearing-assistive device users. CI users typically perform well at basic rhythm (Cooper et al., 2008; Kim et al., 2010), tempo (Kong et al., 2004), and meter (Cooper et al., 2008) perception tasks (although there is evidence that they perform less well for more complex rhythms (Gfeller et al., 2000; Petersen et al., 2012; Jiam and Limb, 2019). In contrast, CI users perform poorly for spectral and spectro-temporal features, such as pitch (Galvin et al., 2007; Cooper et al., 2008), harmony (Brockmeier et al., 2011), melody (Galvin et al., 2007; Zeng et al., 2014), and timbre (Gfeller et al., 2002c; Drennan and Rubinstein, 2008; Nimmons et al., 2008). CI users also have poorer spectral and temporal modulation detection thresholds than normal-hearing listeners (Choi et al., 2018).

HA users have similar spectral and temporal modulation thresholds to normal-hearing listeners (Choi et al., 2018; Looi et al., 2019) and, like CI users, tend not to have deficits with basic rhythm perception (Looi et al., 2019). HA users have been found to have subnormal pitch, melody, and timbre perception (Choi et al., 2018; Looi et al., 2019). However, HA users tend to perform much better than CI users on music perception tasks, such as instrument identification, melody recognition, and pitch discrimination (Gfeller and Lansing, 1991, 1992; Gfeller et al., 1998, 2002a,c; Fujita and Ito, 1999; Leal et al., 2003). It should, however, be noted that there is substantial variance between individual CI and HA users.

Vision plays an important role in music perception for hearing-impaired listeners. Viewing the performer and reading lyrics can increase their musical enjoyment (Gfeller et al., 2000; Looi and She, 2010) and raves targeted at the deaf community frequently include musical visualization. Furthermore, the size of sung musical intervals can be determined when only viewing the singer’s face (without audio), with larger intervals associated with more head movement, eyebrow raising, and mouth opening (Thompson and Russo, 2007; Abel et al., 2016). Viewing a singer’s face with accompanying audio can also bias the perception of pitch interval size (Thompson et al., 2010), with the mouth apparently increasing in significance as audio signal-to-noise ratios become more challenging (Russo et al., 2011). For musical instruments, visual influences have been observed on timbre perception (Saldana and Rosenblum, 1993), as well as on loudness (Rosenblum and Fowler, 1991) and duration (Schutz and Lipscomb, 2007; Schutz and Kubovy, 2009) perception for rhythms.

Several other factors are known to have important influences on music perception for hearing-impaired listeners. For example, the age at which hearing impairment occurred, the amount of residual hearing retained for CI users, and the efficiency of sequential cognitive processing are predictive of pitch and timbre perception (Gfeller et al., 2000, 2008, 2010; O’Connell et al., 2017). Age is also important, with younger CI users listening to music more often and tending to have better timbre perception (Gfeller et al., 2008, 2010; Drennan et al., 2015). More listening hours and musical training have both been linked to higher acuity and music appraisal scores (Gfeller et al., 2002b, 2008, 2010, 2011; Fu and Galvin, 2007; Galvin et al., 2009; Chen et al., 2010; Looi and She, 2010; Driscoll, 2012). However, no strong relationship has been found between perceptual accuracy and music appraisal or enjoyment (Gfeller et al., 2008; Drennan et al., 2015).

### Limits of Haptic Sensitivity Compared to Hearing-Impaired Listening

To establish how haptic stimulation might effectively augment listening, this section compares the sensitivity of the tactile system to the impaired auditory system. First, sensitivity to frequency, intensity, and temporal features will be considered (for a detailed review in the context of speech perception, see Fletcher and Verschuur, 2021).

While frequency discrimination for CI and other hearing-impaired listeners is poorer than for normal-hearing listeners (Moore, 1996; Turgeon et al., 2015), it is better than for haptic stimulation (Goff, 1967; Rothenberg et al., 1977). Because of this poor frequency resolution, several systems for transmitting sound information through haptic stimulation have mapped sound frequency information to location on the skin using an array of haptic stimulators, each triggered by a different pitch or frequency band (Guelke and Huyssen, 1959; Brooks and Frost, 1983; Fletcher et al., 2020c). Using this approach, high-resolution pitch information has been transferred through haptic stimulation (Fletcher et al., 2020c). This could be important for enhancing music perception in hearing-impaired listeners.

The dynamic range of the tactile system at the arm, wrist, and hand is similar to that available to HA users and is around four times larger than that available through electrical CI stimulation (Verrillo et al., 1969; Moore et al., 1985; Zeng and Galvin, 1999; Zeng et al., 2002; Fletcher et al., 2021a, b). CI users are able to discriminate approximately 20 different intensity steps across their dynamic range (Kreft et al., 2004; Galvin and Fu, 2009). For HA users and for haptic stimulation at the arm, wrist, or hand, approximately 40 different steps can be discriminated (Hall and Fernandes, 1983; Gescheider et al., 1996; Fletcher et al., 2021a, b). Interestingly, there is evidence that congenitally deaf people have higher tactile sensitivity than those with normal hearing (Levanen and Hamdorf, 2001), which may mean that the available dynamic range is larger than has been estimated previously in studies using participants with no known hearing impairment. The tactile system therefore seems well suited to deliver sound intensity information to CI users and could provide additional intensity information for at least a subset of HA users.

As highlighted above, CI users typically perform well when extracting temporal sound features. Temporal gap detection thresholds for hearing-impaired listeners and CI users are typically only slightly worse than those for normal-hearing listeners (Moore and Glasberg, 1988; Garadat and Pfingst, 2011). Gap detection thresholds for the tactile system are worse than for most hearing-impaired listeners (Gescheider, 1966, 1967) and tactile signals are more susceptible to masking from temporally remote maskers (Elliot, 1962; Gescheider et al., 1989; Shannon, 1990). Haptic stimulation may therefore not be suitable for providing complex temporal information.

The tactile system has been shown to be highly sensitive to amplitude modulation (Weisenberger, 1986). For a carrier tone at 250 Hz – the frequency at which tactile sensitivity is highest (Verrillo et al., 1969) and a common characteristic frequency for compact motors – amplitude modulation sensitivity was found to be high across the range of frequency modulations most important for speech and music (Drullman et al., 1994; Ding et al., 2017). Sensitivity was reduced when the carrier tone frequency was reduced to 100 Hz (around the lowest characteristic frequency for a compact motor). At modulation frequencies most important to music and speech, amplitude modulation sensitivity for a 250-Hz carrier is below that for an auditory tone carrier at 250 Hz (Zwicker, 1952), but similar to auditory sensitivity for a narrowband noise centred at 200 Hz (Viemeister, 1979), in normal-hearing listeners. This suggests that amplitude modulation is a highly viable route through which sound information can be transferred through haptic stimulation, particularly for CI users, who have reduced sensitivity to amplitude modulation (Choi et al., 2018).

Besides transferring sound information through stimulation at a single site or at adjacent sites, recent studies have shown that sound location information can be transferred through across-limb stimulation (Fletcher and Zgheib, 2020; Fletcher et al., 2020a,2021a, b). CI and HA users have reduced sound localization accuracy compared to normal hearing listeners (Dorman et al., 2016); using this approach, large improvements in sound localization accuracy for CI users were shown, with accuracy reaching levels that could be beneficial to HA users. In this approach, the sound received by devices behind each ear was converted to haptic stimulation on each wrist (Fletcher and Zgheib, 2020; Fletcher et al., 2020a). This meant that time and intensity differences between the ears, which are critical sound localization cues, were available through time and intensity differences across the wrists. Recently, the tactile system has been shown to be highly sensitive to intensity differences across the arms and wrists, but insensitive to time differences (Fletcher et al., 2021a, b). Strikingly, sensitivity to tactile intensity differences across the limbs matched the sensitivity of the auditory system to intensity differences across the ears. Given that instruments in most musical pieces are mapped to a left-right spatial location using only amplitude panning, this high sensitivity to across-limb tactile intensity differences might be exploited to improve localization and segregation of musical instruments.

### Multisensory Integration of Auditory and Haptic Signals

Effective integration of haptic and auditory inputs in the brain is likely to be crucial to haptic augmentation of musical listening. Encouragingly, projections from tactile brain regions have been observed at all stages along the auditory pathway (Aitkin et al., 1981; Foxe et al., 2000; Shore et al., 2000, 2003; Caetano and Jousmaki, 2006; Allman et al., 2009; Meredith and Allman, 2015). Furthermore, physiological studies have shown that the responses of large numbers of auditory cortical neurons can be modulated by input from tactile pathways (Lakatos et al., 2007; Meredith and Allman, 2015) and neuroimaging studies have shown that haptic stimulation can activate auditory cortex (Schurmann et al., 2006); interestingly, stronger activation has been found for deaf participants than for normal-hearing subjects (Levanen and Hamdorf, 2001; Auer et al., 2007). One study in normal-hearing subjects tracked the time course of cortical activation for haptic stimulation on the fingertip (Caetano and Jousmaki, 2006). Initial responses peaked in primary tactile cortical brain regions around 60 ms after the stimulus onset. This was followed by transient responses to the haptic signal in auditory cortex between 100 and 200 ms after onset, before a sustained response was seen between 200 and 700 ms after onset. This could indicate that tactile responses feed forward from tactile brain regions to influence auditory brain regions.

Behavioral studies also offer a range of evidence that haptic and auditory input is integrated. For example, haptic stimulation has been shown to improve sound detection (Schurmann et al., 2004), modulate perceived loudness (Gillmeister and Eimer, 2007; Merchel et al., 2009), and influence syllable perception (Gick and Derrick, 2009). Other studies have shown that tactile feedback from a musical instrument can influence a performer’s perception of sound quality (Fontana et al., 2017). Audio and haptic stimulation have also been effectively combined to improve speech-in-noise performance (Drullman and Bronkhorst, 2004; Huang et al., 2017; Fletcher et al., 2018, 2019, 2020b) and sound localization (Fletcher et al., 2020a).

When considering whether haptic and audio input will be integrated to improve music perception, individual characteristics such as age at which hearing loss occurred, length of time spent with hearing loss, and length of time spent with a hearing-assistive device may be critical. It has been observed that those who receive a CI after a few years of deafness integrate audio and visual information less effectively than those who are implanted shortly after deafness (Bergeson et al., 2005; Schorr et al., 2005; Tremblay et al., 2010). It is possible that a similar limitation will be seen for audio-haptic integration. Some studies have also shown evidence that audio-haptic integration is reduced in congenitally deaf CI recipients compared to late-deafness CI recipients (Landry et al., 2013; Nava et al., 2014). Future work should establish whether benefit of haptic stimulation to music perception is dependent on these factors.

Age may also be important. Haptic stimulation has been shown to improve performance when combined with auditory stimulation in both young (Drullman and Bronkhorst, 2004; Fletcher et al., 2018; Ciesla et al., 2019) and older (Huang et al., 2017; Fletcher et al., 2019, 2020a,b) adults, although these groups have not been directly compared. Several studies have shown evidence that multisensory integration increases in older adults (Laurienti et al., 2006; Rouger et al., 2007; Diederich et al., 2008; Strelnikov et al., 2009, 2015; de Dieuleveult et al., 2017) and there is also evidence that young brains are particularly open to integrating multisensory stimuli (Lewkowicz and Ghazanfar, 2006). It is therefore possible that older adults and children will benefit most from haptic enhancement of music perception.

Auditory deprivation has been associated with increased sensitivity to visual (Finney et al., 2001, 2003) and tactile (Auer et al., 2007) stimuli in auditory brain regions. During early development, substantial neural pruning occurs based on the sensory input received. If auditory input is limited or extinguished by congenital or early-onset deafness, this process can be disrupted and non-auditory inputs can take over auditory brain areas (Quartz and Sejnowski, 1997; Sharma et al., 2007; Glennon et al., 2020). If auditory pathways later receive new sensory stimulation (e.g., because a CI has been fitted), this is thought to compete for neural resources in auditory brain regions with the other sensory inputs that have become established (Sharma et al., 2007; Glennon et al., 2020). This may explain why early implantation is associated with better speech performance (Robbins et al., 2004; Svirsky et al., 2004; Kral, 2009; Tajudeen et al., 2010) and why more visual takeover of auditory brain regions is associated with poorer speech outcomes (Lee et al., 2001; Sandmann et al., 2012; Zhou et al., 2018). The influence of auditory-derived haptic stimulation on this process is unknown, but it may be that such an input would allow auditory brain areas to tune to critical auditory features, such as the amplitude envelope, in the absence of auditory input. Such a process might allow auditory input to compete for neural resources more effectively once input has been restored and might facilitate more effective audio-haptic integration. Future work should explore these possibilities.

Visual input is thought to provide missing speech and sound location information when the audio signal is degraded, to calibrate auditory neural responses, and to guide auditory perceptual learning (Rouger et al., 2007; Bernstein et al., 2013; Strelnikov et al., 2013; Isaiah et al., 2014). As discussed, audio-derived haptic stimulation has been shown to provide missing speech and sound location information when audio is degraded (e.g., Fletcher et al., 2019, 2020a) and to improve lip-reading ability in the absence of auditory stimulation (e.g., De Filippo, 1984; Brooks et al., 1986b; Hanin et al., 1988; Cowan et al., 1991; Reed et al., 1992). However, it has not yet been established whether haptic stimulation can calibrate auditory neural responses or guide auditory perceptual learning. There are relatively few studies of tactile influences on auditory cortex, but one has shown tactile stimulation can enhance responses to auditory input by modulating the rhythm of ambient neural responses in auditory cortex (Lakatos et al., 2007). This might reflect a critical mechanism for haptic enhancement of music perception.

Training is important both for integration of audio and haptic information and for extraction of information from haptic stimulation. Studies with haptic devices for providing speech information when no auditory information is available have shown continued benefits of training throughout long-term training regimes (Sparks et al., 1979; Brooks et al., 1985). Other studies have also shown the importance of training for maximizing haptic sound-localization accuracy (Fletcher and Zgheib, 2020; Fletcher et al., 2020a) and for improving speech-in-noise performance in CI users (Fletcher et al., 2018, 2019, 2020b).

The delay in arrival time of the haptic signal relative to the audio signal is also likely to be important for maximizing integration. A study using broadband signals showed that audio and haptic signals were judged to be simultaneous if the haptic signal onset was delayed from the audio by up to around 25 ms (Altinsoy, 2003). Another study with musical instruments found that the delay at which audio and haptic signal were no longer judged to be simultaneous varied across musical instruments, with attack time seemingly an important factor (Kim et al., 2006). It should be noted that there is significant evidence of rapid temporal recalibration, whereby stimulation from two modalities (including audio and tactile) that are consistently delayed by tens of milliseconds rapidly become perceived as synchronized, provided that they are highly correlated (Navarra et al., 2007; Keetels and Vroomen, 2008; van der Burg et al., 2013). There is evidence that integration occurs even for substantially delayed audio and haptic stimulation. Haptic stimulation has been shown to influence vowel perception, with no statistically significant reduction in this effect when the haptic signal onset was delayed from the audio onset by up to 100 ms (Gick et al., 2010). If haptic signal delays of several tens of milliseconds do not reduce the benefits of haptic stimulation, sophisticated real-time signal-processing strategies could be deployed to enhance music perception.

## Current Systems for Improving Music Perception Using Haptic Stimulation

A range of systems have been developed to enhance music perception using haptic stimulation. At the largest scale, these include systems used for delivering whole-body vibration, such as those used at Deaf Raves, where music containing a lot of low-frequency energy is played at a high intensity. There is evidence that whole-body low-frequency vibration, which is also common during live pop or organ concerts, can play a significant role in the quality of the concert experience (Merchel and Altinsoy, 2014). There is also evidence that vibrating floors can improve the synchronization of dancing to music for hearing-impaired listeners (Shibasaki et al., 2016; Tranchant et al., 2017).

In addition to these large-scale systems, several smaller systems built into chairs have been developed. These typically use a multi-band filtering approach similar to that used in devices to improve access to speech cues in hearing-impaired people (e.g., Brooks et al., 1986a; Fletcher et al., 2019; reviewed in Fletcher, 2020; Fletcher and Verschuur, 2021). In this approach, the audio signal is separated into multiple frequency bands, with each band represented by a haptic stimulator at a different location on the skin. One example is the Emoti-Chair, which has eight haptic stimulators at different body locations (Karam et al., 2009, 2010). Users of the Emoti-Chair were shown to be able to discriminate between a cello, piano, and trombone (matched for fundamental frequency, duration, and intensity), and to be able to discriminate bright from dull timbres (varying only by spectral centroid) (Russo et al., 2012).

**FIGURE 1:**
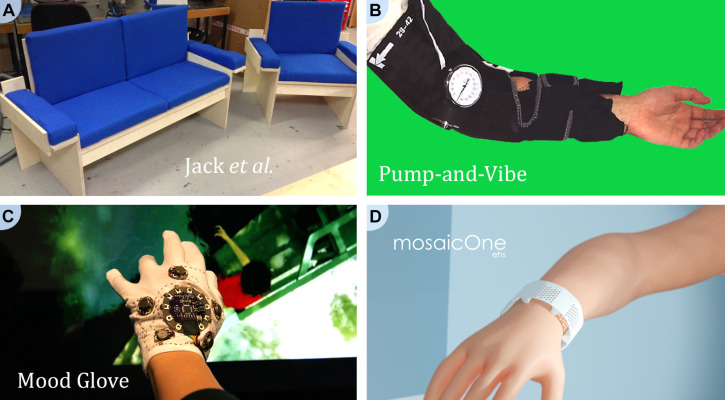
Examples of haptic devices for enhancing music perception. Panel **(A)** Haptic chair developed at Queen Mary University of London (United Kingdom) by Jack and colleagues. Image reproduced with permission of Robert Jack and Andrew McPherson. Panel **(B)** The Pump-and-Vibe, developed at University of Bristol (United Kingdom) by Haynes and colleagues. Adapted from an image reproduced with permission of Alice Haynes. Panel **(C)** The Mood Glove, developed at Queen Mary University of London (United Kingdom) by Antonella Mazzoni. Image reproduced with her permission. Panel **(D)** The mosaicOne_C, developed at the University of Southampton (United Kingdom) by Samuel Perry and Mark Fletcher as part of the Electro-Haptics Research Project. Image reproduced with their permission.

Another chair system developed by Jack et al. (2015) also splits the sound into frequency bands that are mapped to different haptic stimulators (see Figure 1A). In addition to haptic stimulation transferring information about energy within each frequency band, the bandwidth of haptic stimulation at each stimulator is modulated to deliver timbre information (spectral flatness). While subjective reports when using this system were favorable, formal behavioral testing was not performed. They did note, however, that highly rhythmic music tended to be received more positively than music that relied heavily on harmonic motion.

A final example is the haptic chair built by Nanayakkara et al. (2009), which delivered unprocessed music through contact loudspeakers targeting the feet, back, arms, and hands. In their study with 43 young hearing-impaired listeners (with their hearing aids switched off), participants rated their musical experience considerably higher with vibration through the chair than without. However, there were several limitations to the study, including the absence of control for novelty or placebo effects and the possible influence of audio from the contact loudspeakers.

Other medium-scale wearable systems have also been developed, typically deployed using suits or vests. One system uses a suit with 13 haptic stimulators placed around the body and maps different musical instruments to different stimulators (Gunther et al., 2003). A major limitation of this approach is that it requires access to each instrument within a musical piece, which is not typically possible. No formal testing of this haptic suit was performed, although informal feedback from individuals using it as part of an art exhibition was reported to be favorable.

Another wearable system, the LIVEJACKET, which uses a vest with 22 haptic stimulators attached to the arms and torso has also been developed (Hashizume et al., 2018). Like the haptic suit, the LIVEJACKET presents different musical instruments through different haptic stimulators. Survey results suggested the LIVEJACKET enhanced the musical experience for normal-hearing participants. However, critical experimental controls were not in place and, like for the haptic suit, access to each instrument within the musical piece is required.

Finally, there are a range of more compact wearable systems. One such system is the Pump-and-Vibe (Haynes et al., 2021), which is worn on the arm (Figure 1B). The Pump-and-Vibe has eight vibration motors mounted on the forearm arm and an air pump on the upper arm to modulate pressure (“squeeze”). Squeeze is thought to more effectively elicit emotional responses than vibration (Tsetserukou, 2010) and has been deployed in a number of previous devices for various applications (e.g., Chinello et al., 2014; Gupta et al., 2017; Moriyama et al., 2018; Stephens-Fripp et al., 2018; Pezent et al., 2019). The Pump-and-Vibe system aimed to increase the emotional impact of music. The rhythm of the bass was mapped to changes in the amount of squeeze. The squeeze system used was unable to track fast rhythms, so these were mapped to three vibrotactile motors at the top of the forearm. Melody information was mapped to the remaining five motors, with pitch mapped to the location of stimulation along the arm. For vibration, intensity changes were mapped to co-varying haptic frequency and amplitude changes. Sound information was extracted from music using a process involving an online audio-to-MIDI converter. It is not clear how effective this conversion will be for different music types. A qualitative assessment of the Pump-and-Vibe evaluated the mood evoked by a musical piece for audio alone, haptic alone, and haptic and audio together in young participants with no specified hearing impairment (Haynes et al., 2021). Results suggested that the system could evoke moods and influence the mood evoked by audio.

Other examples of more compact systems are the Mood Glove and the mosaicOne series of devices. The Mood Glove (Figure 1C) has eight motors, with five mounted on the back of the hand and three on the palm (Mazzoni and Bryan-Kinns, 2016). Stimulation frequency and intensity are adjusted to portray different moods in musical pieces. A study of the device reported that low-frequency pulses could induce a feeling of calmness and higher-frequency pulses a feeling of excitement (Mazzoni and Bryan-Kinns, 2016). However, the Mood Glove requires the intended mood created by each section of the musical piece to be extracted and provided to the device, which was achieved in the study through manual labeling. This requirement substantially limits the potential for real-world use.

The mosaicOne_B, has two sets of six haptic stimulators arranged along the top and underside of the forearm (Fletcher et al., 2020c). It maps the fundamental frequency of sound (an acoustic correlate of pitch) to location on the skin. Using this device, participants were able to discriminate fundamental frequency differences of just 1.4%. This is markedly better than can be achieved by most CI users (Kang et al., 2009; Drennan et al., 2015) and would allow discrimination of the smallest fundamental frequency changes found in most western melodies. The mosaicOne_B incorporates a novel noise-reduction strategy that was found to be highly effective, with discrimination performance retained even with high levels of background noise. However, it is important to note that the background noise used was inharmonic, while many musical pieces contain multiple simultaneous harmonic sounds. Further work is required to establish the resilience of the mosaicOne_B against harmonic background noise. Furthermore, development is required to allow the device to extract multiple pitches simultaneously, for tracking of multiple simultaneous harmonic instruments. Musical experience was not formally tested using this device, but users reported enhanced musical enjoyment (when listening and feeling pop music) in informal testing by the author of this review with several normal-hearing listeners. Another version of the device, the mosaicOne_C (Figure 1D), has also been developed, which uses a similar approach to that described above, but with shakers spaced around the wrist (Fletcher, 2020; Fletcher and Verschuur, 2021). This device has not yet been subjected to behavioral testing.

Two further studies reported behavioral results for wearable devices. One wrist-worn device extracted the fundamental frequency, like the mosaicOne_B, but mapped it to changes in the frequency and amplitude of the haptic signal (which varied together), rather than spatial location (Luo and Hayes, 2019). Critically, unlike for the mosaicOne_B, this meant that intensity information could not be delivered. Another device delivered the low-frequency portion of the audio signal through haptic stimulation on the fingertip (Huang et al., 2019). Encouragingly, both systems were shown to improve melody recognition. However, the effectiveness of these devices in the presence of background noise has not been tested, and the effect on music appreciation also remains to be established.

In addition to devices developed to augment music perception, several devices have been developed to aid those with sensory impairments by substituting one sense with another. An early example of a sensory substitution device is the Teletactor, developed in the 1920s, which transferred sound to deaf listeners through tactile stimulation on the hand (Gault, 1924, 1926). The principle has since been applied across a number of senses, with systems developed to substitute vision with tactile (Bach-Y-Rita et al., 1969), vestibular with tactile (Bach-Y-Rita et al., 2005), and vision with audio (Meijer, 1992). While these devices have shown promising results, few have found widespread use. Several factors have likely led to this. For example, many systems are highly restrictive, such as the BrainPort (Bach-Y-Rita et al., 2003, 2005) that stimulates the tongue, leaving users unable to speak or eat whilst using the device. Limitations in technology have also often heavily limited discreetness, comfort, and effectiveness. For example, the tactile aids for hearing that were developed in the 1980s and 1990s (before being superseded by CIs (see Fletcher and Verschuur, 2021)) were often large, had short battery lives, and could only perform crude signal processing. However, many of these technological limitations have since been overcome (Fletcher, 2020).

Some of the key design considerations when developing a modern haptic device for enhancing listening are discussed by Fletcher (2020). However, when developing a device for those with hearing-impairment, close engagement with the intended users (such as the deaf community) will be critical for ensuring maximum uptake. Fletcher (2020) advocates a wrist-worn device because they are easy to self-fit, offer a relatively large design space, and because wrist-worn devices, such as smartwatches and exercise trackers, are commonplace and therefore aesthetically acceptable. Indeed, technology for enhancing music perception using haptics could in future be embedded into smartwatches and exercise trackers.

## Haptic Signal-Processing Approaches

Music is commonly accessed through streaming services. This opens the possibility of using signal-processing approaches that cannot be applied in real-time or that are non-causal (require the ability to look ahead). It also opens the possibility of using pre-trained machine-learning algorithms that are selected between based on metadata sent through the streaming service. These algorithms could be trained using the numerous high-quality musical corpora available, which can be supplemented using advanced automated music generation algorithms (Herremans and Chuan, 2020). So-called “near real-time” algorithms, which have processing delays of no more than a few seconds, may be of particular interest as such a delay before playback might be tolerable if clear enhancement of music experience could be demonstrated. Nevertheless, since a substantial portion of music is not streamed (e.g., at a concert or as background music in a shop), real-time signal-processing approaches are still preferred. Current evidence suggests that large delays of haptic stimulation from audio stimulation might be tolerable, which would allow sophisticated real-time signal-processing approaches to be deployed (see section “Multisensory Integration of Auditory and Haptic Signals”). Both real-time and offline approaches should therefore be considered.

It is important to first establish the goal when converting audio to haptics for music enhancement. One approach is to remove elements that reduce clarity when audio is transferred at a low-resolution (e.g., through a CI). One example of this is spectral complexity reduction, in which the frequency spectrum is sparsened and simplified, using methods such as principal component analysis (Nagathil et al., 2017; Gauer et al., 2019). Spectrally reduced musical pieces have been shown to be preferred for CI listening (Nagathil et al., 2017) and a similar approach might be trialed for haptic enhancement of music perception. An alternative approach is to enhance perception of certain instruments within a multi-instrument piece. It has been observed that CI and HA users find musical pieces with multiple instruments less pleasant than pieces with a single instrument (Looi et al., 2007) and that CI users prefer pop music with the vocal level substantially increased (Buyens et al., 2014). It may therefore be desirable to separate instruments and use haptic stimulation to enhance one or a small subset.

### Source Separation

Some basic methods for separating sound sources have already been used for converting audio to haptic stimulation. One haptic signal-processing approach uses an expander, which amplifies loud sounds, to extract speech from background noise when the signal-to-noise ratio (SNR) is positive (i.e., the speech is louder than the noise; Fletcher et al., 2018, 2019). This simple real-time approach improves speech-in-noise performance for CI users at positive SNRs but is not expected to be suitable for enhancing music, where the SNRs for individual instruments are typically less favorable. Another approach used pitch extraction methods to separate harmonic and inharmonic sounds (Fletcher et al., 2020c). Pitch extraction is often susceptible to background noise (Jouvet and Laprie, 2017), but the proposed approach was shown to be robust to inharmonic noise (Fletcher et al., 2020c). However, this and other pitch extraction approaches for enhancing music perception using haptics (e.g., Luo and Hayes, 2019), are not designed to accommodate musical pieces with multiple simultaneous harmonic sounds. More advanced multi-pitch extraction methods will likely be required if they are to be effective across a range of musical pieces.

A range of noise-reduction techniques are deployed in hearing-assistive devices to extract speech from background noise, and these might also have utility for haptic signal-processing strategies. One commonly used group of techniques focus on the temporal domain. These exploit the fact that the amplitude envelope of speech tends to have a lower modulation frequency and depth than environmental noise (Ding et al., 2017; Lakshmi et al., 2021). These techniques classify speech signals as having a modulation rate less than around 10–30 Hz and a modulation depth greater than around 15 dB (e.g., Schum, 2003). Another commonly used group of techniques focus on the spectral domain. These estimate the spectrum of the background noise and subtract this from the speech-in-noise signal. To determine when only background noise is present, these spectral subtraction techniques typically employ a voice detector (Boll, 1979; Ephraim and Malah, 1984). Another approach, that is less commonly used in modern hearing-assistive devices, focuses on harmonic structure. Unlike many noise signals, speech contains harmonics with strong co-modulation. Synchrony detection algorithms classify the signal as speech if it has highly synchronous energy fluctuations across frequency bands (Schum, 2003). The latest noise-reduction strategies in hearing-assistive devices often deploy multiple noise-reduction approaches, as well as using environmental classification methods and adaptive filtering (Ricketts and Hornsby, 2005; Peeters et al., 2009). These techniques might be adapted to focus on the typical characteristics of musical instruments (e.g., Ding et al., 2017), although it should be noted that these approaches were developed to extract a single sound source and that musical instruments often share temporal and spectral characteristics. Furthermore, a recent meta-analysis found no significant improvement in speech intelligibility with digital noise-reduction algorithms in HA users, although subjective outcomes, such as sound quality, did show moderate improvement (Lakshmi et al., 2021).

Many HAs have dedicated signal-processing settings for music listening. While manufacturers often do not reveal exactly how these differ from those for improving speech-in-noise performance, they often appear to reduce or remove the noise-reduction applied and use slower-acting compression (Moore, 2016). In a survey of HA users, no clear difference in music experience was found between those with a dedicated music setting on their HA and those without (Madsen and Moore, 2014).

More advanced methods for separating sound sources in musical pieces have also been developed. One approach attempts to separate harmonic and percussive sounds (Buyens et al., 2014, 2015). While this approach may have utility for haptic signal-processing, its potential is significantly limited by the fact that it cannot separate common key instruments, such as vocals and bass, from each other. Another method using non-negative matrix factorization has shown potential for separating and enhancing vocals, although notable distortions and artifacts were observed (Pons et al., 2016). More advanced machine-learning-based source separation methods have also been tested and were found to outperform non-negative matrix factorization (Gajecki and Nogueira, 2018). Deep convolutional auto encoders, which combine denoising auto encoding and convolutional neural networks, performed extremely well, but only when the audio processed was similar to that used to train the algorithm. Multilayer perceptrons and deep recurrent neural networks, on the other hand, performed well across a range of data. The authors concluded that multilayer perceptrons were most suitable because they were faster to compute, although none of the techniques tested were implemented in real-time. A recent study developed a real-time multilayer perceptron method, which was shown to be effective in isolating vocals and to be robust to background noise and reverb that would be encountered with live audio (Tahmasebi et al., 2020). Advanced source separation approaches like these could be critical to maximizing the effectiveness of haptic devices for enhancing music perception.

### Feature Extraction

In addition to deciding the source or sources to be separated, it will be important to determine which sound features should be provided through haptic stimulation. Features shown to enhance speech perception when presented through haptic stimulation, such as amplitude envelope (e.g., Brooks and Frost, 1983; Fletcher et al., 2019) and fundamental frequency (e.g., Huang et al., 2017), should be explored. The utility of other features, like those used by the Moving Picture Expert Group for audio content, should also be investigated as they could provide additional information, such as timbre (as in, for example, Jack et al., 2015). These include: spectral features, such as centroid, spread, and flatness; harmonic features, such as centroid, spread, variation, and deviation; and temporal features, such as centroid and log attack time (see Zhang and Ras, 2007).

The optimal features to extract are likely to differ across instruments and musical styles. For example, vocals in rap music might require rhythmic information through features such as amplitude envelope, whereas vocals in show tunes may benefit more from pitch-based features, such as fundamental frequency. For a non-harmonic instrument like a snare drum, pitch-based features cannot be extracted and features like spectral spread or spectral centroid might be most appropriate.

Sound classification algorithms will be important to any approach that selects features based on instrument type or musical style. A range of methods for music genre classification have shown promise, including ensemble classifiers and methods that implement sound source segregation approaches, such as non-negative matrix factorization (Silla et al., 2007; Pérez-García et al., 2010; Rosner and Kostek, 2018). Several instrument classification approaches have also shown promise, including advanced methods using deep convolutional neural networks (Benetos et al., 2006; Gomez et al., 2018; Solanki and Pandey, 2019; Racharla et al., 2020). Establishing the most effective classification approaches and auditory features to provide through haptic stimulation will be a critical part of future research in this area.

### Haptic Mapping

Having separated the instruments and extracted sound features, the next consideration will be how to map these to haptic stimulation. Haptic music-enhancement approaches should take advantage of the tactile system’s large dynamic range (Verrillo et al., 1969; Fletcher et al., 2021a, b) and high sensitivity to intensity differences, both at a single site and across sites (Gescheider et al., 1996; Fletcher et al., 2021a, b). As discussed (see section “Limits of Haptic Sensitivity Compared to Hearing-Impaired Listening”), this might include spatially mapping instruments using amplitude panning across sites, such as the wrists (Fletcher and Zgheib, 2020; Fletcher et al., 2020a, b), that mimics amplitude panning of instruments within a musical piece. Stimulus features (such as fundamental frequency) might also be mapped to changes in spatial location on the skin to maximize information transfer (e.g., Brooks and Frost, 1983; Karam et al., 2010; Fletcher et al., 2020c).

## Important Cutting-Edge Technologies

Modern haptic devices can take advantage of critical recent advances in technology (see Fletcher, 2020 for a detailed review). These include: haptic motor and driver technology to deliver high-fidelity stimulation with low power usage; battery technology, to increase the potential power usage and reduce the necessity for frequent charging; manufacturing techniques, such as 3D printing, to facilitate the development of comfortable, aesthetically acceptable, and easy to self-fit devices; wireless technologies, to allow audio streaming from remote microphones and other devices and to link processing across multiple stimulation points on the body; and microprocessors to allow advanced signal-processing. Future devices might also take advantage of flexible microprocessor technology, which is currently being developed (Biggs et al., 2021). This could allow additional signal-processing capacity to be built into device components that need to be flexible, such as straps.

**FIGURE 2:**
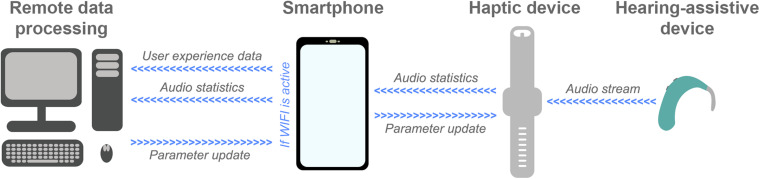
Schematic representation of an example future remote data-processing pipeline for haptic signal-processing optimization (described in the text). Audio is streamed from a hearing-assistive device to a haptic device that extracts audio statistics and sends them to a smartphone. A smartphone app also collects user feedback about their experience using the haptic device. Audio statistics and user experience data are then sent for remote data processing, where optimized signal-processing parameters are determined. Finally, these updated parameters are sent to the smartphone and uploaded to the haptic device.

Several other recent and ongoing technological developments could be exploited to maximize haptic enhancement of music perception. One example is big data systems that have the capacity to collect data from devices as they are being used in the real world. This technology is currently being exploited in the EVOTION platform (funded by the European Union) and the HearingFitness program (developed by Oticon Medical), which use big data collected from devices in the real world to inform policy-making (Gutenberg et al., 2018; Dritsakis et al., 2020; Saunders et al., 2020). In future, the technology might also be used to optimize haptic signal-processing. Figure 2 shows an example remote data processing pipeline. In this pipeline, audio is streamed to the haptic device from a hearing-assistive device to ensure maximum correlation between the audio and haptic signals (see Fletcher, 2020). Audio statistics, such as spectral flatness and short-term energy, are then extracted by the haptic device and transferred to a smartphone. The smartphone also has an app to collect user feedback, for example ratings of sound quality and music enjoyment, and to link clinical data such as hearing-assistive device type and hearing-loss profile. Audio statistics and user data are stored on the smartphone and uploaded to a remote server or The Cloud when a WIFI connection is established (to reduce power consumption and mobile data usage). The data is processed remotely to update models and derive optimized signal-processing parameters. These models could be optimized for each individual or be used as part of a big data approach for optimizing signal-processing globally, for subgroups of users, or for different music types. Once updated signal-processing parameters are determined, these are transferred to the haptic device via the smartphone.

To implement a remote data processing pipeline of this sort, exploitation of cutting-edge technology and further research are required. It should be noted that, in practice, simpler systems that collect user feedback to optimize new iterations of algorithms might be developed before a full pipeline like that proposed is implemented. One key technology for the proposed pipeline is wireless data streaming. This can be achieved using the latest Bluetooth Low Energy technology, which allows multiple simultaneous data streams, has low power usage, and is already integrated into many of the latest hearing-assistive devices. Another critical element is the development of a smartphone app for collecting user feedback, which must have a high level of data security and privacy. User feedback is likely to be important as music perception varies substantially across hearing-impaired listeners due to factors such as previous musical experience (Galvin et al., 2009; Gfeller et al., 2015). The app developed for the proposed system can build on existing apps that are already deployed in the growing field of telemedicine to collect real-world user feedback for optimization of hearing-assistive devices, such as ReSound Assist (Convery et al., 2020). Finally, future research will be required to determine the optimal audio statistics to be extracted and sent for remote processing, as well as the most effective approaches for processing this data and deriving optimal signal-processing parameters. The recent expansion in remote data collection and analysis capacity through systems such as Cloud computing will be critical in allowing big data to be processed with sophisticated models.

In addition to user- and stimulus-based optimization of signal processing, steps should be taken to ensure that haptic stimulation is perceived as uniformly as possible across users. One simple way to do this is to adjust the stimulation intensity based on each user’s detection thresholds (as is done for hearing-assistive devices). It may also be important to adapt the intensity based on the fitting of the device on the body. The fitting (e.g., how tightly the device is strapped on) can substantially alter the amount of pressure applied to the haptic motor and the coupling with the skin. Techniques have recently been developed to estimate the pressing force on the motor and dynamically calibrate it (Dementyev et al., 2020). Such techniques should be explored for future haptic devices for enhancing music perception.

## Discussion

Music perception is often significantly impaired in those with hearing loss. Critical factors are the loss of ability to discriminate sounds of different frequencies and a reduction in dynamic range. Recently, it has been shown that haptic devices can be highly effective at providing intensity (Fletcher and Zgheib, 2020; Fletcher et al., 2020a, 2021a,b) and frequency information (Fletcher et al., 2020c), and can support perception of complex signals such as speech (Huang et al., 2017; Fletcher et al., 2018, 2019, 2020b). However, despite the large number of haptic systems that have been developed for enhancing music perception, there is a lack of robust data on whether haptic devices can effectively improve music perception for hearing-impaired listeners. Whilst haptic stimulation has vast potential to enhance music perception, a significant research program is required to provide a clear evidence base.

Several critical technologies have been developed in recent years, which can be exploited in future haptic devices. These allow faithful haptic signal reproduction, advanced signal processing, wireless communication between hardware components (such as smartphones, microphones, and haptic devices), long battery lives, and rapid prototyping and manufacturing. These technologies give scope for vast improvements to current haptic devices for enhancing hearing. In addition, several key emerging technologies and methods have been identified, which further expand the potential for haptic enhancement of music perception. These include cloud computing and cutting-edge machine-learning approaches. Exploitation of these new technologies could considerably increase haptic enhancement of listening and allow a dramatic expansion in access to music and other media for hearing-impaired listeners.

Another consideration raised in this review is the interaction between haptic, audio, and visual stimulation. It was highlighted that significant sound information from music is accessible through vision, particularly pitch interval size and direction. Future work should establish whether critical sound information, such as pitch, provided through haptic, audio, and visual modalities can be effectively combined to enhance discrimination. It will also be critical to explore how providing sound information through non-auditory senses can alter auditory perception. This could determine whether future research on haptic enhancement aims to restore conventional music perception or whether it instead seeks to offer an alternative way to experience music.

In addition to enhancing music listening, there is significant potential for haptics to be used for enhancing musical performance in hearing-impaired individuals. Of particular interest might be enhancement of vocal performance. CI users often have considerable difficulties when singing, particularly in producing the correct pitch (Xu et al., 2009; Mao et al., 2013). There have been some promising results when providing pitch information to hearing-impaired listeners through haptic stimulation to improve singing (Sakajiri et al., 2010, 2013; Shin et al., 2020; Hopkins et al., 2021). Future work should establish the effectiveness of the alternative pitch-based haptic stimulation approach suggested by Fletcher et al. (2020c), which was shown to provide high-resolution pitch information. These pitch-based approaches might also be highly effective for speech rehabilitation. Congenitally deaf individuals often struggle to acquire and maintain normal speech (Smith, 1975; Gold, 1980), and those who suffer hearing loss later in life often also experience a reduction in vocal control, often including greater pitch variability (Lane and Webster, 1991).

This review has discussed the enormous potential of haptic stimulation to enhance music listening. It is estimated that around 1.6 billion people across the world have hearing loss, with this number expected to increase rapidly (Haile et al., 2021). Alongside this growth in the number of people who need support with hearing impairment is a rapid growth in technologies that could improve and expand this support. The use of haptic stimulation to enhance listening for those with hearing impairment offers an opportunity to exploit many of these recently developed technologies. The time therefore seems right for a major expansion of research into haptic enhancement of listening.

If effective and accessible systems are developed, as well as directly enhancing music enjoyment, they could substantially improve access to and enjoyment of media (such as films and documentaries), video games, and social events, such as weddings. Furthermore, given that music is an extremely challenging signal because of its complexity, progress in this area could have substantial benefits for enhancing communication and spatial awareness in complex everyday acoustic environments. Thanks to inexpensive core technologies, haptic devices could become widely accessible, including in low- and middle-income countries, and bring substantial improvements in quality of life for those with hearing impairment.

## Author Contributions

The author confirms being the sole contributor of this work and has approved it for publication.

## Conflict of Interest

The author declares that the research was conducted in the absence of any commercial or financial relationships that could be construed as a potential conflict of interest.

## Publisher’s Note

All claims expressed in this article are solely those of the authors and do not necessarily represent those of their affiliated organizations, or those of the publisher, the editors and the reviewers. Any product that may be evaluated in this article, or claim that may be made by its manufacturer, is not guaranteed or endorsed by the publisher.
